# Coffee polyphenols ameliorate early-life stress-induced cognitive deficits in male mice

**DOI:** 10.1016/j.ynstr.2024.100641

**Published:** 2024-05-15

**Authors:** J. Geertsema, M. Kratochvil, R. González-Domínguez, S. Lefèvre-Arbogast, D.Y. Low, A. Du Preez, H. Lee, M. Urpi-Sarda, A. Sánchez-Pla, L. Aigner, C. Samieri, C. Andres-Lacueva, C. Manach, S. Thuret, P.J. Lucassen, A. Korosi

**Affiliations:** a Center for Neuroscience, Swammerdam Institute for Life Sciences, University of Amsterdam, 1098 XH, Amsterdam, the Netherlands; b Biomarkers and Nutrimetabolomics Laboratory, Food Innovation Network (XIA), Nutrition and Food Safety Research Institute (INSA), Faculty of Pharmacy and Food Sciences, University of Barcelona, 08028, Barcelona, Spain; c CIBER Fragilidad y Envejecimiento Saludable (CIBERfes), Instituto de Salud Carlos III, 28029, Madrid, Spain; d University of Bordeaux, Inserm, Bordeaux Population Health Research Center, UMR 1219, F-33000, Bordeaux, France; e Université Clermont Auvergne, INRAE, UNH, F-63000, Clermont Ferrand, France; f Department of Basic and Clinical Neuroscience, Maurice Wohl Clinical Neuroscience Institute, Institute of Psychiatry, Psychology and Neuroscience, King's College London, London, SE5 9NU, UK; g Department of Genetics, Microbiology and Statistics, University of Barcelona, 08028, Barcelona, Spain; h Institute of Molecular Regenerative Medicine, Spinal Cord Injury and Tissue Regeneration Center Salzburg, Paracelsus Medical University, Salzburg, 5020, Austria

**Keywords:** Early-life stress, Polyphenols, Microglia, Cognition

## Abstract

Stress exposure during the sensitive period of early development has been shown to program the brain and increases the risk to develop cognitive deficits later in life. We have shown earlier that early-life stress (ES) leads to cognitive decline at an adult age, associated with changes in adult hippocampal neurogenesis and neuroinflammation. In particular, ES has been shown to affect neurogenesis rate and the survival of newborn cells later in life as well as microglia, modulating their response to immune or metabolic challenges later in life. Both of these processes possibly contribute to the ES-induced cognitive deficits. Emerging evidence by us and others indicates that early nutritional interventions can protect against these ES-induced effects through nutritional programming. Based on human metabolomics studies, we identified various coffee-related metabolites to be part of a protective molecular signature against cognitive decline in humans. Caffeic and chlorogenic acids are coffee-polyphenols and have been described to have potent anti-oxidant and anti-inflammatory actions. Therefore, we here aimed to test whether supplementing caffeic and chlorogenic acids to the early diet could also protect against ES-induced cognitive deficits. We induced ES via the limited nesting and bedding paradigm in mice from postnatal(P) day 2–9. On P2, mice received a diet to which 0.02% chlorogenic acid (5-O-caffeoylquinic acid) + 0.02% caffeic acid (3′,4′-dihydroxycinnamic acid) were added, or a control diet up until P42. At 4 months of age, all mice were subjected to a behavioral test battery and their brains were stained for markers for microglia and neurogenesis. We found that coffee polyphenols supplemented early in life protected against ES-induced cognitive deficits, potentially this is mediated by the survival of neurons or microglia, but possibly other mechanisms not studied here are mediating the effects. This study provides additional support for the potential of early nutritional interventions and highlights polyphenols as nutrients that can protect against cognitive decline, in particular for vulnerable populations exposed to ES.

## Abbreviations

BBB: Blood brain barrier
BrdU: 5-bromo-2′-deoxyuridine
CA: Cornu ammonis
CTL: Control
DG: Dentate gyrus
ES: Early-life stress
HCPC: Hierarchical clustering of principle components
ITI: Intertrial interval
LBN: Limited bedding and nesting
ML: Molecular layer
MWM: Morris water maze
MS: Maternal separation
OLT: Object location task
ORT: Object recognition task
P: Postnatal day
PB: Phospate-buffered
PBS: Phosphate-buffered saline
PCA: Principal component analysis
PFA: Paraformaldehyde
Poly: Diet containing 0.02% chlorogenic acid and 0.02% caffeic acid
PPS: Postpartum stress
PUFAs: Polyunsaturated fatty acids
ROI: Region of interest
RT: Room temperature
SEM: Standard error of the mean
SGZ: Subgranular zone
SLM: Stratum lacunosum moleculare
Std: Standard
TBS: Tris-buffered saline
v: Ventral

## 1 Introduction

Exposure to stress during early-life is associated with an increased risk to develop cognitive impairments and psychopathologies later in life (Short and Baram, 2019; Carr et al., 2013; Naninck et al., 2015). This association is confirmed by preclinical studies in which ES led to cognitive impairments which were accompanied by specific alterations in brain plasticity, including adult hippocampal neurogenesis (Naninck et al., 2015; Baek et al., 2011; Lajud et al., 2012; Mirescu et al., 2004; Oomen et al., 2009; Lucassen et al., 2015; Korosi et al., 2012) and neuroinflammation (Hoeijmakers et al., 2014, 2017; Bachiller et al., 2022; Lumertz et al., 2022; Reemst et al., 2022a). In particular, we and others have shown earlier that the survival of newborn cells (Naninck et al., 2015) and of other stages of adult hippocampal neurogenesis are reduced by ES (Baek et al., 2011; Lajud et al., 2012; Oomen et al., 2009). Concerning neuroinflammation, ES further affects the ‘macrophages of the brain’, i.e. the morphology, transcriptome and phagocytic capacity of microglia, as well as their response to later life challenges (e.g. LPS administration) (Hoeijmakers et al., 2017; Reemst et al., 2022a; Delpech et al., 2016; Diz-Chaves et al., 2013; Dayananda et al., 2023). Given their functional roles, the ES-induced alterations in hippocampal neurogenesis and microglia could potentially contribute to the observed cognitive deficits later in life (Green and Nolan, 2014).

While there has been great progress in understanding the neurobiological processes contributing to the ES effects on cognition later in life, effective interventions for this vulnerable population are currently still lacking. There is evidence that early nutritional interventions could prevent the ES-induced changes in behavior (Arabi et al., 2021; Donoso et al., 2020; Naninck et al., 2017; Yam et al., 2019; Juncker et al., 2022). For example, we have shown in a limited bedding and nesting model (LBN) that ES led to impairment in spatial memory and object recognition tasks in males, but not females (Naninck et al., 2015) and that early supplementation with early micronutrients (P2-9) or N-3 polyunsaturated fatty acids (PUFAs) from P2-42 protected against these ES-induced cognitive deficits later in life (Naninck et al., 2017; Yam et al., 2019). In particular, these beneficial effects of N-3 PUFA supplementation were associated with a rescue of the ES-induced alterations in the survival of newborn cells and neuroinflammation (Yam et al., 2019).

Recently, also other potential candidates for nutritional interventions have surfaced. In fact, in an untargeted metabolomic prospective cohort study (spanning 12 years of cognitive assessment follow-up), coffee-metabolites, which are markers of consumption of coffee, were identified to be protective against cognitive decline in humans (Low et al., 2019; González‐Domínguez et al., 2021). This raised our interest in the potential protective effects of more bioactive compounds, such as coffee-derived polyphenols including chlorogenic and caffeic acid. Therefore, we here aim to further investigate the potential of these polyphenols, and test whether they can be protective against cognitive decline in ES-exposed male mice.

Polyphenols are plant metabolites naturally occurring in many fruits, vegetables, coffee, tea, and wine (Quideau et al., 2011) and their protective potential in the context of psychiatric- and cognitive disorders has been suggested before (Morris et al., 2021; Gomez-Pinilla and Nguyen, 2012; de Vries et al., 2021). Although the exact mechanisms of action of these compounds and their microbial metabolites in the brain are not fully understood, they have demonstrated to be able to positively affect cerebrovascular blood flow, protect the blood-brain barrier (BBB) and to mitigate neuroinflammation through the modulation of diverse intracellular signalling pathways and the regulation of transcription factors such as Nuclear factor-κ B, Nuclear factor erythroid 2-related factor 2, and Aryl hydrocarbon receptor, resulting in particular in an inhibition of the release of cytokines and other pro-inflammatory mediators, as well as a reduction of reactive oxygen species generation in response to microglia activation (Corral-Jara et al., 2021; Pinto et al., 2023; Carregosa et al., 2021; Vauzour, 2017). An effect through the intestinal barrier protection has also been suggested (Wang et al., 2022).

There is further evidence that magnolia polyphenols attenuate oxidative- and inflammatory responses in neurons and microglial cells (Chuang et al., 2013) and that flavonoids extracted from Xiaobuxin-Tang reverse decreases in hippocampal neurogenesis and neurotrophic molecule expression in chronically stressed rats (An et al., 2008), suggesting that polyphenols might exert their beneficial effects via modulation of microglia and hippocampal neurogenesis. These properties are specifically interesting in the context of ES as the neuro-immune system (Hoeijmakers et al., 2017; Bachiller et al., 2022; Lumertz et al., 2022) and neurogenesis (Naninck et al., 2015) have been reportedly affected by ES. There is also evidence that polyphenols (ferulic acid (Zheng et al., 2019) and kolaviron (Omotoso et al., 2020)) protect against behavioral alterations induced by ES (in the form of prenatal stress (Zheng et al., 2019) and maternal deprivation (Omotoso et al., 2020)) when administered after the stressor (P60-88) and (P21-35) respectively.

Here, we study whether polyphenols derived from coffee (0.02% chlorogenic acid and 0.02% caffeic acid, equivalent to 2 cups of coffee) supplemented early in life could protect against chronic ES-induced effects on cognition in mice. We focus on male mice due to their increased vulnerability to cognitive decline when exposed to LBN (Naninck et al., 2015). In addition, we study whether this dietary modulation could be mediated by newborn cell survival or microglia. We show that the ES-induced cognitive decline is prevented by an early diet (P2-42) supplemented with chlorogenic- and caffeic acid and that the modulation of microglia, but not newborn cell survival, might contribute to these effects.

## 2 Material and methods

### 2.1 Animals, ES model and diet

C57BL/6 mice were bred in-house to control for perinatal environmental conditions, as described previously (Naninck et al., 2015). If a nest was detected between 9 a.m. and 10 a.m., P0 was assigned to the day before. Dams and her pups were exposed to postpartum stress (PPS) and ES respectively, using the LBN paradigm from P2-9, as previously described (Naninck et al., 2015; Rice et al., 2008). Briefly, at P2, nests were culled to 5–6 pups keeping the sex-ratio as equal as possible, followed by random assignment to either stress-control ((CTL): 100 g bedding, 1 piece of nestling material; ES: 33 g bedding, half a nestlet, stainless steel grid mesh placed 1 cm above bedding) and both diet conditions. Dams were introduced to either a grain-based diet (AIN-93G, Ssniff-Spezialdiäten GmbH, Soest, Germany) containing 0.02% chlorogenic acid (5-O-caffeoylquinic acid) + 0.02% caffeic acid (3′,4′-dihydroxycinnamic acid) (Poly), or a standard chow diet (Std) matched to this diet from P2-42. Our diets (both experimental and control diet) were grain-based diets, as synthetic diets may lack essential fibers, which in turn could lead to compromised gut health and systemic inflammation, potentially influencing outcome measures (Pellizzon and Ricci, 2020; Tuck et al., 2020).

**Fig. 1:**
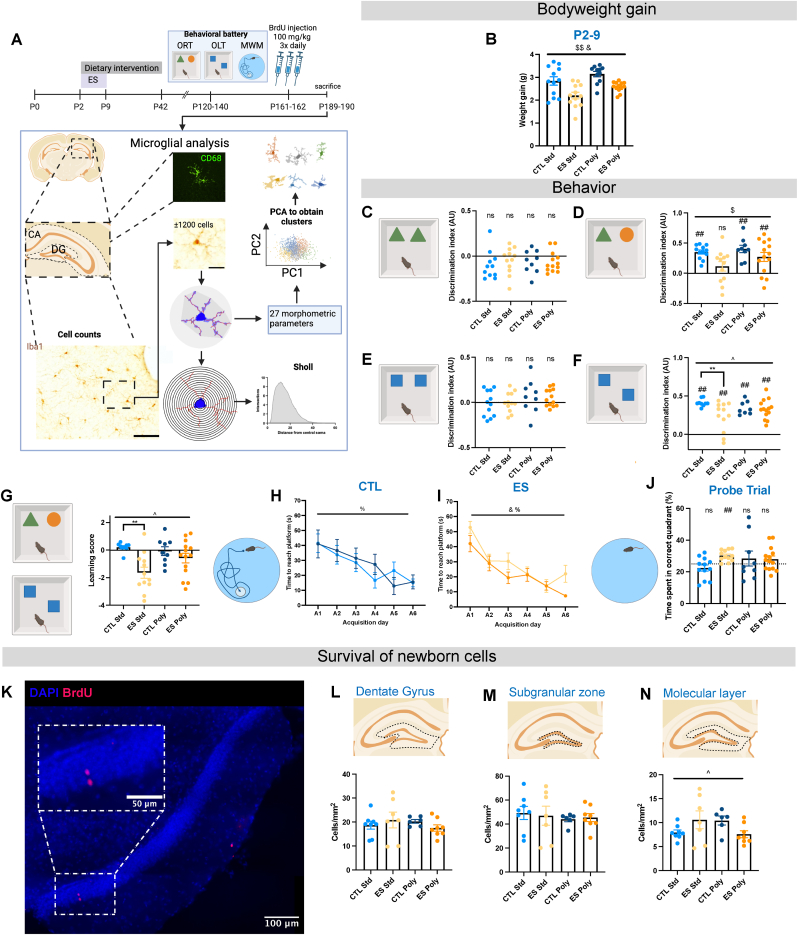
**– Experimental workflow, and ES and diet effects on bodyweight, behavior and survival of newborn cells.** A) Experimental design. Mice were exposed to ES using LBN from P2-9. At the same time, from P2-42, a polyphenol diet or Std diet was administered. At P120, mice underwent behavioral testing. Three weeks later, mice were injected with BrdU for 3 times per day for 2 days, and four weeks later, animals were sacrificed. B) ES decreased bodyweight gain between P2–P9, while diet increased bodyweight gain. C) Discrimination index during the training phase of the ORT was not statistically significant from 0 for all experimental groups. D) ES impaired discrimination between novel and familiar objects during the testing phase of the ORT, which was reversed by supplementation with the polyphenol diet. In general, ES animals discriminated less between novel and familiar objects. E) Discrimination index during the training phase of the OLT was not statistically significant from 0 for all experimental groups. F) All groups discriminated between novel and familiar objects during the testing phase of the OLT. On the Std diet, ES animals discriminated less between novel and familiar objects, but this was not the case on the polyphenol diet. G) ES animals had a lower learning score on the Std diet, but not on the polyphenol diet. H) CTL animals reached the platform more quickly over time on both diets. I) ES animals reached the platform more quickly over time, and Polyphenol diet fed animals performed better than the Std fed animals. J) ES animals fed the Std diet spent more time in the target quadrant compared to other quadrants during the probe trial, all other groups failed to perform better than chance. K) Representative stitched overlay image (40x) of BrdU (red) staining and DAPI (blue) of (part of) the DG for CTL Std animals, dashed lines indicate a zoom-in of that area. L) ES and diet did not affect survival of newborn cells in the DG. M) Stress and diet did not affect survival of newborn cells in the SGZ. N) There was an interaction of diet and ES in their effect on survival of newborn cells in the ML. Annotations: For two-way ANOVAs % is day of acquisition effect p < 0.05, $ is ES effect p < 0.05, & is diet effect p < 0.05, ^ is interaction effect ES x Diet p < 0.05, * is post hoc Tukey effect p < 0.05; for one-sample *t*-test # is mean is different from 0 (DI) and 25 (probe trial) p < 0.05, ns is not significant p > 0.05. All values are Data ±SEM.

Male mice were weaned at P21 and littermates were group-housed (2–4 animals per cage). At P42, all mice were switched back to standard chow (CRM(P)). Mice were weighed at the start and end of the ES-paradigm and dietary intervention, at weaning, and then monthly until sacrificed. Before animals were weaned, the weight increase of an individual animal versus the average weight of animals in a cage was calculated. Food intake was measured throughout the dietary intervention up until one week after. Food intake by the dams exposed to PPS was recorded from P2-9 and from P9-21 and corrected for litter size. Total food intake by pups exposed to ES was corrected for the number of cage mates. Animals were further left undisturbed until an age of P120 ± 15 days. For an overview of the experimental design, see Fig. 1A. All experimental procedures were executed according to the Dutch national law and European Union directives on animal experiments and were approved by the animal welfare committee of the University of Amsterdam.

### 2.2 Behavioral observations and testing

Male mice (CTL Std n = 12; ES Std n = 12; CTL Poly n = 9; ES Poly n = 14) were subjected to a behavioral test battery including the object recognition task (ORT), the object location task (OLT) and the Morris water maze task (MWM). During testing, the mice were recorded by a video camera connected to a computer, set up with Ethovision software. The ORT and OLT videos were manually scored by an experimenter blind to the conditions using Observer XT 9.0 (Noldus Information Technology). Two weeks before the first behavioral test starting around four months of age, the mice were housed in a room with a reversed dark/light schedule (12/12 h light/dark - lights on at 8 p.m.). All mice were handled for 5 min for five consecutive days prior to behavioral testing. During testing, mice were brought into the testing room 1 h prior to the start of the habituation, training, or testing. All behavioral testing was done during the dark (active) phase. There was a one-day interval between the ORT, the OLT and the MWM. Because the behavioral experiments were performed in two batches, we corrected for batch effects when necessary, using a linear mixed model.

#### 2.2.1 Object recognition task

Prior to the ORT, animals were habituated for 5 min every day for three days to the testing arena (transport box L x B = 34.8 × 27.5 cm), which was covered with sawdust. Subsequently, during the training phase, mice were left to freely explore two identical objects for 5 min. After an intertrial interval (ITI) of 24 h, one of the objects was replaced by a novel object and mice were again left to freely explore both objects for 5 min and the time that the head of the animal was in close proximity to the objects was scored and the discrimination index calculated (see 2.2.2.1).

#### 2.2.2 Object location task

Mice were re-habituated to the testing arena for 5 min in one day. Two identical objects (but distinct from the objects used for the ORT) were placed in the testing arena. During the training, animals were allowed to freely explore both objects. The next day, after an ITI of 24 h, one object was moved to a novel location in the testing arena. The mice were reintroduced to both objects for 5 min and again scored for the time that the head of the animal was in close proximity to the objects was scored and the discrimination index calculated (see 2.2.2.1) *2.2.2.1 OLT and ORT analyses*.

Analysis was done in the same way for the ORT and the OLT. For both the training and testing phase of the ORT and OLT, we assessed the discrimination index (For training: (total exploration time object 1 – total exploration time object 2)/(total exploration time object1 + object 2), for testing (total exploration time novel object – total exploration time familiar object)/(total exploration time novel object + total exploration time familiar object)), which is used as an index of memory. An index significantly above 0 indicates a preference for the novel object. The mean discrimination index of each group was compared to zero by performing a one-sample *t*-test. To assess stress and diet effects on the DI among experimental groups, we performed a two-way ANOVA. During training, when mice had a cumulative exploration time of less than 10 s, the animal was excluded from analysis.

#### 2.2.3 Morris water maze

During habituation, the mice were placed in the centre of a circular maze (diameter: 100 cm) filled with water (20–23 ֯C) with a platform in one of the quadrants and allowed to explore for 60 s. The water level was as such that the platform rose 1–2 cm above the water, visible for the mice. The habituation phase lasted one day with two trials and an ITl of 30 min.

Before the acquisition phase started, water was opacified by non-toxic, white paint. Spatial cues were placed on the walls encircling the pool and the platform was now placed 1 cm below the water surface in the northwest-quadrant. Animals were allowed to swim from starting point to the platform for a maximum of 60 s, to prevent fatigue. An ego-centric search strategy was prevented by randomly varying the starting point between trials. The acquisition phase lasted six days with two trials and an ITI of 30 min. For each trial, the latency towards entrance of the platform was measured.

At the end of the acquisition phase, the platform was removed for the probe trial. During the probe trial (which lasted 60 s), the time spent in the correct quadrant was scored. During the probe trial, the percentage of time spent in the target quadrant was used as an index of memory: a percentage significantly above 25 (chance level) indicates a preference for the quadrant where the platform used to be and is used as a proxy for memory. For the reversal phase, the platform was brought back into the pool in the southeast-quadrant and animals were tested for cognitive flexibility. In a trial, animals were allowed to swim from the starting point to the platform for a maximum of 60 s. The reversal phase lasted four days with two trials and an ITI of 30 min. For each trial, the latency to reach the platform was measured.

### 2.3 BrdU injections

Mice were injected intraperitoneally three times a day for two days, with 5-bromo-2′-deoxyuridine (BrdU; 100 mg/kg, Sigma-Aldrich), at three weeks after behavioral testing, and four weeks before sacrifice to ensure that the behavioral tasks did not interact with the BrdU incorporation.

### 2.4 Tissue collection and dissection

Four weeks after BrdU injections, the mice were anaesthetized with an injection of Euthasol (AST Farma, 100 mg/kg) and transcardially perfused with saline (0.09% NaCl in Aquadest), followed by 4% paraformaldehyde (PFA). The perfused brains were post-fixed in 4% PFA for 24 h before moving them to Phospate-buffered (PB)-azide for temporary storage. Before sectioning, brain tissue was cryoprotected by moving them first to a 15%, then a 30% sucrose solution in PB at 4 °C. Using a sliding microtome, all brain tissue was coronally sectioned at a thickness of 40 μm in 6 parallel series and then stored in anti-freeze (30% Ethylene glycol, 20% Glycerol, 50% 0.05 M phosphate-buffered saline (PBS)) at −20 °C before use.

### 2.5 Immunohistochemistry and imaging

#### 2.5.1 BrdU staining and imaging

BrdU staining was performed in four batches with an equal representation of each condition (4 × 8 = 32 animals) per day (total animal numbers: CTL Std n = 8; ES Std n = 8; CTL Poly n = 8; ES Poly n = 8). We tested for batch effects and included batch as a factor when necessary in a linear mixed model. Sections were first mounted on glass slides and antigen retrieval was performed to improve the immunoreactive signal: sections were first exposed to 2 N HCl for 20 min at 37 ֯C in an oven and afterwards washed for 10 min in 0.1 M Na_2_B_4_O_7_. The sections were then quickly washed with PBS (0.05 M, 0.9% saline; pH 7.4) before being incubated for 2 h in blocking solution (3% bovine serum albumin (BSA), 0.30% Triton X-100 in PBS).

Subsequently, the sections were incubated overnight at 4 ֯C with the primary antibody in blocking solution (Rat anti-BrdU, Accurate Chemical and Scientific Corporation OBT0030, 1:500). Subsequently, the sections were rinsed for 5 × 5 min with washing solution (PBS-T: 0.25% Triton X-100 in 0.05 M PBS) before getting incubated with secondary antibodies (Donkey anti-Rat Alexa fluor 488, Invitrogen Thermo Fisher Scientific, 1:500; and DAPI, Invitrogen Thermo Fisher Scientific, 1:1000; in blocking solution) for 2 h on the shaker at RT in the dark. The incubation was followed by a quick wash with PBS and slides were then covered with embedding medium containing DAPI and coverslipped.

Three stained dorsal (Bregma points −1.06 to −1.94) and three stained ventral (Bregma points −1.94 to −3.80) sections of the dentate gyrus (DG) were imaged (Nikon eclipse H600L fluorescent microscope 40× objective, stitched image (size depended on bregma point), NIS-Elements BR 4.60.00 64-bit software).

#### 2.5.2 Iba1 staining and imaging

Free-floating sections (total animal numbers: CTL Std n = 8; ES Std n = 8; CTL Poly n = 6; ES Poly n = 8) were being washed 3 × 5 min in 0.05 M Tris-buffered saline (TBS) (pH 7.6) and subsequently treated in 0.3% hydrogen peroxide in TBS for 15 min to block endogenous peroxidase activity. This was followed by washing in TBS 3 × 5 min and a blocking step of 30 min in blocking mix (1% BSA + 0.3% triton-x in TBS). The sections were incubated for 1 h at room temperature (RT) followed by an overnight incubation at 4 °C with primary antibody in the blocking mix (polyclonal rabbit anti-Iba1 (a general microglia marker), Wako, 1:1000). The day after, sections were washed in 0.05 M TBS +0.3% Triton X-100 (3 × 5 min) and this was followed by an incubation with a secondary antibody (biotinylated goat anti-rabbit (Vector), 1:500) for 2 h. Following a second wash in 0.05 M TBS (3 × 5 min), sections were incubated in avidin-biotin complex in 0.05 M TBS for 90 min (ABC Elite kit, Vectastain, Brunschwig Chemie, Amsterdam, the Netherlands, 1:800 for each component). After washing sections for 5 min in 0.05 M TBS and subsequently, in 0.05 m tris buffer (TB) (4 × 5 min), sections were incubated for 20 min with 0.5 mg/ml 3,3′-diaminobenzidine (DAB) in 0.05 M TB (pH = 7.6) with 0.1% H_2_O_2_. Sections were fast washed three times in TB, followed by washes in TB (2 × 5 min). After mounting the sections on a slide, they were dried overnight, dehydrated, and covered with Entallan.

Two dorsal and two ventral hippocampal sections were imaged (Nikon eclipse H600L widefield microscope 20× objective, 2×2 stitched image, LED 15%, NIS-Elements BR 4.60.00 64-bit software) to distinguish the DG from the cornu ammonis (CA) of the hippocampus.

#### 2.5.3 CD68 staining and imaging

Free-floating sections (total animal numbers: CTL Std n = 8; ES Std n = 8; CTL Poly n = 8; ES Poly n = 8) were washed in 0.05 M TBS for 3 × 5 min, and blocked with a solution of 3% BSA and 0.3% triton-x in TBS. Following this, sections were incubated with primary antibody (CD68 - MCA 1957, rat anti-mouse monoclonal, Serotec AbD, 1:500) in an incubation mix of 1% BSA and 0.3% triton-x in TBS overnight at 4 ֯C. After primary AB incubation, sections were washed in TBS for 3 × 5 min and incubated with secondary antibody (A-11077, goat anti-rat Alexa fluor 568, Invitrogen, 1:500) in incubation mix for 2 h at RT. Finally, sections were washed in TBS for 3 × 5 min, placed in 0.05 M tris buffer, mounted on slides, and coverslipped with mounting medium containing DAPI.

Two dorsal and two ventral sections were imaged using a confocal microscope (Nikon A1; 20× objective, 6×2 stitched image with 20 x 1μ Z steps), imaging a section containing both the DG and CA of the hippocampus.

### 2.6 Quantifications

#### 2.6.1 Volume estimations

To estimate the volume of the hippocampus, the DG and the subgranular zone (SGZ), the Cavalieri principle was employed as described previously (Hoeijmakers et al., 2017). A surface estimation (in mm^2^) of the DG at each level was obtained using the DAPI staining, by tracing the structures in Fiji software in six sections per animal. The area was then multiplied by six as sections were split into six series, and then multiplied by the thickness of sections (0.04 mm). As we used six out of nine hippocampal sections per series, we multiplied by 9/6.

#### 2.6.2 BrdU+ and Iba1+ counts

Quantification of Iba1+ and BrdU + cell numbers and densities was done by an experimenter blinded to the experimental groups. BrdU + cells were manually counted in regions of interest (ROIs) of the whole DG, consisting of the hilus, the SGZ and the molecular layer (ML). The amount of BrdU + cells in each of these regions were added up and corrected for the surface area of the DG (calculated earlier). Iba1+ cells were manually counted in ROIs of the CA and DG. When looking at hippocampal cell density, Iba1+ cells were manually counted in all ROIs from four images (Four ROIs per regions CA and DG), added up and divided by the area of all ROIs and data are presented as BrdU+ and Iba1+ cells per surface area.

#### 2.6.3 Coverage analysis

Images were converted to 8-bit black-and-white images and ROIs were drawn based on the DAPI signal to outline the DG and the CA. A fixed threshold, equal for all experimental conditions was determined for the CD68 staining, after which the percentage of immunoreactive stained area (coverage) in the respective regions could be calculated. CD68 coverage was corrected for cell density in each experimental condition.

#### 2.6.4 Complexity analysis

1200 representative Iba1+ cells were selected from images by an experimenter blind to experimental groups. For both the CA and DG, we extracted 20 cells per region per animal, derived from both dorsal and ventral slices, representing the full hippocampus. The single-cell images were processed to cell silhouette images by local thresholding and subjected to automated skeletonization, Sholl- and fractal analyses and 27 morphometric parameters were extracted as described before (van Weering et al., 2023). A principal component analysis (PCA) was applied on 23 of these parameters, which had an eigenvalue of >1 to reduce the influence of high collinearity. Subsequently, a hierarchical clustering on principal components (HCPC) in all cells was conducted to identify 6 different microglial morphotypes. To assess whether general cluster distribution was different among experimental conditions, a MANOVA was conducted. Subsequently, the effects of ES and diet were tested in each cluster with an alpha of 0.05/6 (six clusters). Separate ANOVAs have been performed per independent parameter, and the effects of ES and diet with an alpha of 0.05/17. The Sholl plots were shown for visualization. From these plots, the area under the curve was calculated per cell and plotted for all four conditions. The number of primary branches was one of the morphometric parameters that contributed to the clustering of cells and is a measure for complexity.

### 2.7 Statistical analysis

Data were analyzed using R (version 4.2.1) and visualized with GraphPad Prism 9 and expressed as mean ± standard error of the mean (SEM). Data were considered statistically significant when p < 0.05 (or, when multiple testing was in place, alpha is clearly stated in Supplementary Table 1. Data points that were outside the 1.5 interquartile range were deemed as outliers and excluded from analysis. Assumptions of parametric analysis were tested using the Shapiro-Wilk normality test and Levene's test for homogeneity of variance. When data could be transformed to follow a normal distribution, this was done to use a parametric test, otherwise non-parametric statistic tests were performed, see Supplementary Table 1.

Because multiple animals from one litter were included in this study, we always tested for possible contributing effects of litter by performing a mixed model analysis with litter as a random factor. When the model significantly improved including nest number, it was added and a linear mixed model was performed (see Supplementary Table 1). When a linear mixed model was performed, residuals were inspected visually in R. In addition, because multiple cells from one animal were sampled for the analysis of AUC and primary branching, we averaged these data points/animal resulting in one datapoint per animal for each measure.

## 3 Results

### 3.1 Effects of ES, diet and their interaction on bodyweight gain and food intake

Bodyweight was measured at P2, P9, P21, P42, and at 2, 3 and 4 months of age (Supplementary Fig. 1A). Overall, there was a significant effect of condition, diet, and age on bodyweight gain and food intake (Repeated-Measures Three-way ANOVA: Condition F (1,43) = 4.729, p < 0.05; Diet F (1,43) = 4.955, p < 0.05; Age GGe (3.18, 136.6) = 0.529, p < 0.01). More specifically, ES reduced the weight gain between P2 and P9, corroborating our previous findings (Naninck et al., 2015; Reemst et al., 2022a). Moreover, animals on the polyphenol diet had an increased bodyweight gain compared to the animals fed the Std diet (Fig. 1B Two-way ANOVA: Condition F (1, 45) = 18.607, p < 0.01; Diet F (1, 45) = 6.716, p < 0.05).

In the time following ES, until weaning (P9-21), no significant differences in bodyweight gain were detected between groups (Supplementary Fig. 1B). At P42, the average weights of all groups were similar, regardless of condition or diet (Supplementary Fig. 1C). Food intake by the dams from P2-9 and P9-21 was unaffected by PPS or diet (Supplementary Figs. 1D–E). After weaning, food intake was similar among all groups of pups at ages P21-42 (Supplementary Fig. 1F). The return to the Std diet did not lead to changes in food intake compared with animals that had always been fed the Std diet. Food intake was also unaffected by ES (Supplementary Fig. 1G).

### 3.2 Effects of ES, diet and their interaction on behavior

To test the effect of ES and early diet and their interaction on later life cognitive functioning, we assessed behavior in the ORT, OLT and MWM. There were no differences among groups in locomotor activity during the habituation phase and cumulative exploration time during the training phase (Supplementary Figs. 2A–B).

To test the effects of ES and early supplementation with polyphenols on recognition memory, we performed an ORT. In the training phase of the ORT, none of the animals exhibited a preference towards one of the objects (Fig. 1C). However, during the testing phase, ES Std animals did not prefer the novel object over the familiar objects, while all other experimental groups did (Fig. 1D One sample *t*-test H_0_: DI = 0: CTL Std t (Bachiller et al., 2022) = 10.712, p < 0.01; ES Std t (Bachiller et al., 2022) = 1.445, p = 0.176; CTL Poly t (Lucassen et al., 2015) = 6.257, p < 0.01; ES Poly t (Reemst et al., 2022a) = 3.617, p < 0.01), suggesting a cognitive deficit in ES animals fed the Std diet in the ORT. Moreover, ES exposed animals spent significantly less time with the novel object compared with the familiar object during the testing phase (Two-way ANOVA: Condition F (1,43) = 7.830, p < 0.01).

To assess whether spatial memory was affected by ES or diet, we exposed mice to OLT and MWM testing. During the training phase of the OLT, none of the animals exhibited a preference between the objects (Fig. 1E). During the testing phase, all animals were able to discriminate between the novel and familiar object (Fig. 1F One sample *t*-test H_0_: DI = 0: CTL Std t (Lucassen et al., 2015) = 25.321, p < 0.01; CTL Poly t (Oomen et al., 2009) = 11.308, p < 0.01; ES Std t (Bachiller et al., 2022) = 4.289, p < 0.01; ES Poly t (Reemst et al., 2022a) = 9.769, p < 0.01). However, despite the fact that all mice learned the task, there was a significant interaction effect of ES and diet among groups (Two-way ANOVA: Condition x Diet F (1,43) = 4.649, p < 0.05). Post hoc tests revealed that ES Std animals explored the novel object less than the than CTL Std animals (p < 0.01), a difference not evident when comparing CTL Poly and CTL Std animals (p = 0.725). When looking at a learning score (i.e., composing one Z-score for both tasks), we see a strong detrimental effect of ES under the Std diet, which is not significant when comparing the polyphenol supplemented groups (Fig. 1G Linear mixed model: Condition x Diet t (Bachiller et al., 2022) = -2.380 p < 0.05, CTL Std - ES Std p < 0.01 and CTL Poly – ES Poly p > 0.05). Concerning the MWM, all animals, independent of diet or condition, acquired the task both when looking at short and long-term memory by exploring the first and second trial of each day separately, or when the trials were averaged (Supplementary Figs. 2C–E Repeated Measures Three-way ANOVAs combined: Day GGe (3.79, 159.02) = 0.757, p < 0.01; first trial: Day GGe (3.74, 153.48) = 0.749, p < 0.05; second trial: Day GGe (4.18, 153.48) = 0.836, p < 0.01). To further explore potentially different effects of the diet between experimental groups, we also analyzed the CTL and ES groups separately. When analyzing the acquisition times in the second trial, we found that diet did not affect acquisition time in CTL groups, but polyphenol supplementation reduced acquisition time in ES-exposed mice (Fig. 1H–I Repeated Measures Two-way ANOVA: Diet F (1,120) = 4.473, p < 0.5).

During the probe trial, only the ES animals fed the Std diet performed significantly better than chance (Fig. 1J; One sample *t*-test H_0_: Time spent in target quadrant = 25: CTL Std t (Bachiller et al., 2022) = −1.325, p = 0.212; CTL Poly t (Lucassen et al., 2015) = 0.712, p = 0.497; ES Std t (Bachiller et al., 2022) = 5.202, p < 0.01; ES Poly t (Reemst et al., 2022a) = 1.559, p = 0.143). We found the same pattern during the first 30 s of the probe trial (Supplementary Fig. 2F; One sample *t*-test H_0_: Time spent in target quadrant = 25: CTL Std t (Hoeijmakers et al., 2017) = −1.014, p = 0.335; CTL poly t (Oomen et al., 2009) = 0.282, p = 0.786; ES Std t (Bachiller et al., 2022) = 3.376, p < 0.01); ES Poly t (Reemst et al., 2022a) = 0.359, p = 0.725). Animals fed the polyphenol diet, independent of ES exposure, had a shorter latency towards entering the correct quadrant (Supplementary Fig. 2G, Linear mixed model: Diet t (Tuck et al., 2020) = 2.262 p < 0.05). During the probe trial of the MWM, there were no differences in distance moved among groups, ruling out the influence of possible swimming locomotor activity differences (Supplementary Fig. 2H).

We were additionally interested to see how cognitive flexibility was affected by ES and early polyphenol administration. During reversal learning, acquisition time was not affected by condition or diet or by day of training (Supplementary Fig. 2I).

Given that we observed ES-induced impairments in the ORT and OLT as well as in a composite learning score, which were rescued by the polyphenol diet and that in the MWM, the polyphenol diet improved acquisition specifically in the ES-exposed group, we next aimed to investigate the associated alterations in relevant forms of brain plasticity related to neuronal survival and microglia.

### 3.3 Effects of ES and diet on hippocampal volume and BrdU + cells

The estimated volume of the hippocampus, the DG or the SGZ was not affected by ES or diet (data not shown, test statistics in Supplementary Table 1). We tested the effects of ES and diet on survival of newborn cells by quantifying the amount of BrdU + cells per mm^2^, corrected for volume (for a representative image see Fig. 1K). In the DG and SGZ, no effects of ES or diet were found (Fig. 1L-M), while in the ML there was a significant interaction between diet and ES (Fig. 1N Linear mixed model: Condition x Diet t (Yam et al., 2019) = 2.444, p < 0.05, no significant post hoc tests).

### 3.4 Effects of ES and diet on microglial density, complexity and CD68 expression

Next, we tested whether ES and a polyphenol diet affected Iba1+ cell density and complexity and CD68 expression. We quantified immunoreactive Iba1 cells via; i) manual counts in representative areas of the DG, CA and hippocampus and ii) via detailed unbiased morphological characterization, as performed before (van Weering et al., 2023).

**Fig. 2:**
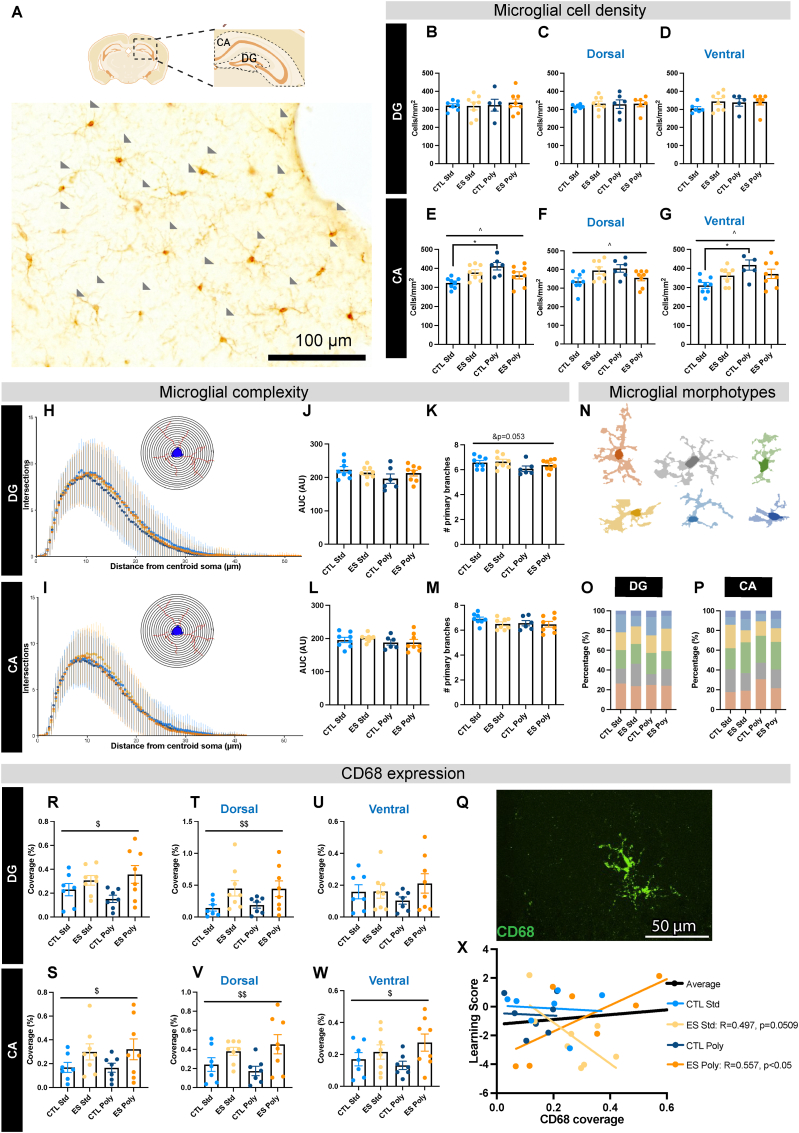
**Effects of diet and ES on microglial cell density, complexity and expression of CD68.** A) Area marked in the dashed boxes are the ROIs. Representative stitched brightfield image (20x) of DAB staining of Iba1 of the hippocampus for each condition. B) There was no effect of ES or polyphenol diet on Iba1+ cell density in the DG. C) There was no effect of ES or polyphenol diet on Iba1+ cell density in the dDG. D) There was no effect of ES or polyphenol diet on Iba1+ cell density in the vDG. E) The polyphenol diet increased Iba1+ cell density in the CA of CTL animals, but not in ES animals. F) There was an interaction of diet and stress on microglial cell density in the dCA. G) The polyphenol diet increased Iba1+ cell density in the vCA density in CTL animals, but not in ES animals. H) Amount of intersections plotted against the distance from the soma of DG Iba1+ cells. I) Amount of intersections plotted against the distance from the soma of CA Iba1+ cells. J) ES and diet did not affect the AUC of the DG Sholl plots. K) Diet tends to reduce number of primary branches of DG Iba1+ cells, independent of ES. L) There was no effect of ES or diet on the AUC of the CA Sholl plots. M) There was no effect of ES or diet on the number of primary branches of CA Iba1+ cells. N) Representative silhouette images of Iba1+ cell clusters, categorized in color per morphotype. O) There was no effect of stress or diet on relative morphotype proportions in the DG. P) There was no effect of stress or diet on relative morphotype proportions in the CA. Q) Cropped image from a representative stitched confocal image (40x) CD68 (green) staining. R) ES increased CD68 coverage in the DG, independent of diet. S) ES increased CD68 coverage in the CA, independent of diet. T) ES increased CD68 coverage in the dDG, independent of diet. U) No effect of stress or diet on CD68 coverage in the vDG. V) ES increased CD68 coverage in the dCA, independent of diet. W) ES increased CD68 coverage in the vCA. X) In ES animals, CD68 coverage correlated with learning score, but not in CTL animals. There was a negative correlation between learning score and CD68 coverage in ES animals fed the Std diet, but a positive correlation in ES animals fed the polyphenol diet. Annotations: For two-way ANOVAs $ is ES effect p < 0.05, & is diet effect p < 0.05, ^ is interaction effect ES x Diet p < 0.05, * is post hoc Tukey effect p < 0.05. All values are Data ±SEM

There was an interaction effect between diet and ES on Iba1+ cell density (for a representative image, see Fig. 2A) when taking the whole hippocampus in account, while these effects disappear when looking at the dorsal and ventral hippocampus separately (Supplementary Figs. 3A–C Two-way ANOVA: Condition x Diet F (1,24) = 6.252, p < 0.05). When looking at the DG and the CA separately, we find region-specific effects. We found no effects of ES and diet on Iba1+ cell density in the DG (Fig. 2B–D), but we did find an interaction effect between diet and ES in the CA (Fig. 2E–G). Specifically, we found an interaction between ES and diet, with the polyphenol diet significantly increasing the number of Iba1+ cells in CTL animals, but not in ES animals (Fig. 2E Two-way ANOVA: Condition x Diet F (1,24) = 10.712, p < 0.01, CTL Std – CTL Poly p < 0.01; ES Std – ES Poly p = 0.894). A similar pattern was found throughout the dorsal(d)CA axis of the CA (Fig. 2F dCA Two-way ANOVAs: Diet x Condition F (1,24) = 9.064, p < 0.01), and the ventral(v)CA (Fig. 2G vCA Two-way ANOVA: F (1,24) = 5.361, p < 0.01), though only in the vCA post hoc testing revealed that the polyphenol diet significantly increased Iba1+ cell counts in CTL, but not ES animals (CTL Std – CTL Poly p < 0.01; ES Std – ES Poly p = 0.988).

To obtain a more general overview of Iba1+ cell morphology, Sholl plots were made for the individual Iba1+ silhouettes per experimental group (Supplementary Fig. 3D for the hippocampus, Fig. 2H for the DG and Fig. 2I for the CA). The polyphenol diet and ES exposure did not affect the area under the curve (AUC) and the number of primary branches in the hippocampus (Supplementary Figs. 3E–F). Subregional analyses revealed no differences in AUC (Fig. 2J), but a trend towards a diet effect on primary branches, where the Poly diet seems to reduce the number of primary branches in the DG (Fig. 2K; Two-way ANOVA: Diet F (1,26) = 4.100, p = 0.053) but not in the CA (Fig. 2L and M).

From the thresholded silhouettes, 27 different parameters could be extracted of which the 23 parameters that were not colinear were subjected to PCA (Supplementary Fig. 3G, Two-way ANOVAs for each parameter can be found in Supplementary Table 1). The distribution of all Iba1+ cells on the first component plane was visualized. PC1 accounted for 50.4% of the variance in the dataset, while PC2 accounted for 14.5% of the variance in the dataset (Supplementary Fig. 3H). Based on these PCAs, an unsupervised HCPC analysis identified six Iba1+ morphotypes (Fig. 2N). Cluster distribution was similar among all 4 experimental groups, in the whole hippocampus (Supplementary Fig. 3I), and in the DG (Fig. 2O) and the CA (Fig. 2P) subregions.

As a proxy for microglial phagocytosis, we investigated the effects of early polyphenol diet and ES on CD68 coverage (Fig. 2Q for a representative image of the staining), which was corrected for the amount of Iba1+ cells per mm^2^. ES increased CD68-expression independent of diet in the hippocampus, DG, and the CA (Supplementary Fig. 3J-L and Fig. 2R and S (Two-way ANOVAs: hippocampus condition F (1,26) = 8.224, p < 0.01; DG Condition F (1,26) = 6.618, p < 0.05); CA Condition F (1,26) = 4.695, p < 0.05). There were dorsoventral differences in the DG and CA. In the DG, the effect of ES was only detected dorsally (Fig. 2T; Two-way ANOVA: Condition F (1,26) = 8.291 p < 0.01), but not ventrally (Fig. 2U), while in the CA, CD68 coverage was increased across both the dCA and the vCA (Fig. 2V-W; Two-way ANOVAs dCA Condition F (1,25) = 8.765, p < 0.01; vCA Condition F (1,25) = 4.653 p < 0.05).

To further investigate the relationship between microglia and cognitive functions, we explored the correlation of hippocampal Iba1+ cell density and CD68 coverage with the learning score. While we did not detect a correlation between Iba1+ cell density and learning scores (Supplementary Fig. 3M), there was a correlation of CD68 coverage with learning scores, which was dependent on experimental group. In mice exposed to ES and supplemented with polyphenols, learning scores were positively correlated with CD68 coverage, while there was a trend towards a negative correlation when ES mice were fed the Std diet (Fig. 2X Spearmans correlations: ES Poly R^2^ = 0.557, p < 0.05; ES Std R^2^ = 0.497, p = 0.051). There was no significant correlation in CTL mice.

## 4 Discussion

In this study, we report that supplementation of an early diet with coffee polyphenols from P2-42 in ES-exposed (P2-9) male mice, protected them against ES-induced later life cognitive deficits. To study possible underlying mechanisms that could mediate these effects, we investigated processes most affected in our previous studies using this same model, i.e. survival of newborn cells and neuroinflammation (Naninck et al., 2015, 2017; Reemst et al., 2022a; Yam et al., 2019). Newborn cell survival was not affected by ES or diet in the DG, but an interaction between ES and polyphenol diet was detected in the ML. Moreover, the polyphenol diet increased microglial cell density in the CA and reduced microglial complexity in the DG, while ES increased microglial CD68 expression throughout the hippocampus without any further effect of the diet. The observed microglial alterations potentially contribute to the alleviating effect of the coffee polyphenol diet on the ES-induced cognitive defects.

### 4.1 Diet supplemented with coffee polyphenol early in life protects against ES-induced cognitive impairments

Coffee polyphenol supplementation early in life and beyond (P2-42) protected against the ES-induced cognitive deficits. We observed ES-induced impairments only in mice in the CTL diet group in the ORT as well as in a composite learning score, while not in mice exposed to the polyphenol diet. In the MWM, the polyphenol diet improved acquisition specifically in the ES-exposed group. Despite the fact that mice from all group acquired the task, only the mice from the ES Std group spent significantly more time in the target quadrant during the probe trial. We currently have no explanation for why our control mice did not learn the tasks. Based on a meta-analysis on 400 independent experiments, it is clear that variation in the behavioral effects of ES can be expected (Bonapersona et al., 2018), and the general consensus is that ES impairs non-stressful learning. Polyphenols have been shown to protect against ES-induced behavioral deficits before in rats (Arabi et al., 2021; Donoso et al., 2020; Omotoso et al., 2020; Menezes et al., 2017). For example, supplementing with auraptene (Arabi et al., 2021), phlorotannins, quercetin or xanthohumol (Donoso et al., 2020), kolaviron (Omotoso et al., 2020) and catechins (Menezes et al., 2017), after maternal separation (MS, i.e. a different ES paradigm), led to improvements in ES-induced anxiety (Arabi et al., 2021; Donoso et al., 2020; Omotoso et al., 2020), depressive-like symptoms (Donoso et al., 2020) and cognitive impairment (Omotoso et al., 2020; Menezes et al., 2017). All of the abovementioned polyphenols have different plant origins, and our study adds to this evidence by showing that also coffee-related polyphenols can modify ES effects, but now in mice subjected to the LBN model. Moreover, we provide evidence for long-term beneficial effects of this early and relatively short supplementation with polyphenols. In contrast to our design, which consisted of an early dietary intervention that ended several months prior to behavioral testing, previous studies in rats started their intervention shortly after the stress paradigm, with the diet being supplemented throughout life, and thus the nutrients could still be present when the rats underwent behavioral testing up until sacrifice (Arabi et al., 2021; Donoso et al., 2020; Omotoso et al., 2020; Menezes et al., 2017).

Taken together, despite the heterogeneity of studies on diets supplemented with polyphenols given during different time windows in specific ES paradigms and species, polyphenols seem promising nutrients in counteracting ES-induced behavioral changes. Next to the beneficial effects of an early polyphenol diet, as described here, the selection of the early life period has proven to be a successful time window for nutritional intervention in the context of ES. Also, other nutrients that were administered during this period were shown to be beneficial, including a combination of micronutrients (Naninck et al., 2017) and N-3 PUFAs (Yam et al., 2019), highlighting the importance for this sensitive developmental period as a critical time window to provide preventive nutritional strategies (Juncker et al., 2022).

### 4.2 Neurobiological processes potentially contributing to the protective effect of coffee polyphenols

#### 4.2.1 Hippocampal volume and survival of newborn cells

The volumes of the hippocampus, the DG and SGZ were unaffected by ES exposure, which is in line with our previous studies (Naninck et al., 2017; Yam et al., 2019). While in clinical studies ES has been shown to reduce hippocampal gray matter volume (Hakamata et al., 2022), in rodent studies, the effect of ES on hippocampal volume is more subtle (Walker et al., 2017). For example, hippocampal volume in adulthood was reduced in mice exposed to LBN (Naninck et al., 2015), while after MS, a decrease in hippocampal volume was observed only up until P30 (Herpfer et al., 2012). Our current findings further underline the subtle nature of the effect of ES on hippocampal volume.

Newborn cell survival was not affected by ES or diet in the DG as a whole, but an interaction between ES and diet was detected in the ML. This contrasts with earlier evidence showing that ES lead to a reduction in the birth and survival of newborn neurons in adulthood (Naninck et al., 2015; Baek et al., 2011; Lajud et al., 2012; Mirescu et al., 2004; Oomen et al., 2009; Lucassen et al., 2015; Korosi et al., 2012). In particular, we had shown before that ES reduced the number of BrdU + cells, which were all NeuN+ (Naninck et al., 2015), which is why we in the current study focused on BrdU + cells only. When assessing the ML and SGZ separately, and thereby distinguishing cells that have and have not migrated further in the hippocampus, we found an interaction of diet and ES in the ML of the hippocampus, suggesting an increased survival of newborn cells after ES. The origin of this discrepancy might lie in the subregional analyses that were performed here rather than taking the full DG into account.

The polyphenol diet led to increased cell survival in CTL mice, while it reversed the ES-induced increase in cell survival. It is intriguing that polyphenols seem to exert a neurogenic effect in CTL mice that is opposite to its effect in ES-exposed mice. Similar to the neurogenic effect of the diet in CTL mice, other studies investigating polyphenols in the context of stress found a stimulatory effect on factors related to neurogenesis. For example, xanthohumol supplementation from postnatal week 8–16 increased brain-derived neurotrophic factor expression in MS-exposed rats in week 16 (Donoso et al., 2020). In a model of prenatal stress, ferulic acid supplementation for four weeks (P60-88) increased the amount of Nissl + bodies (a proxy for neuronal number) in the hippocampus at P95 (Zheng et al., 2019). Four weeks after a period of 28 days, during which chronic unpredictable stress at an adult age was combined with flavonoid supplementation, an increase in hippocampal BrdU + cells was found (An et al., 2008). In our current study we did not detect an ES-induced reduction in neurogenesis, which differs from some previous studies and there are several key differences between them, including the heterogeneity in polyphenols supplemented, the different stress paradigms and timelines, and also the behavioral outcomes measured. However, other nutrients, such as N-3 PUFAs, were able to modulate BrdU + cells lasting into adulthood, when supplemented early in life, notably with a similar timeline as the one used in the current study, suggesting different pathways of action of PUFAs and polyphenols (Yam et al., 2019). While the lack of an ES-induced reduction in DG volume remains unclear, this finding is in line with the lack of effect on cell survival of newborn cells in the DG as well as with the milder effect of ES in hippocampal-dependent learning tasks, compared to our earlier reports (Naninck et al., 2015, 2017; Yam et al., 2019). In addition, we cannot exclude that other stages of neurogenesis are affected by polyphenols, which remains to be investigated in future studies.

#### 4.2.2 Microglia

As ES affects the neuroimmune system in rodents (Hoeijmakers et al., 2017; Reemst et al., 2022a; Delpech et al., 2016; Diz-Chaves et al., 2013; Dayananda et al., 2023; Yam et al., 2019) and polyphenols have anti-inflammatory capacities (Chuang et al., 2013; Zheng et al., 2019; Omotoso et al., 2020), we were interested in studying whether microglia modulation might be involved in the protective effect of polyphenols on the ES-induced cognitive deficits. We show that both ES and polyphenol supplementation individually affect microglia-related parameters, without interaction effects between diet and ES exposure.

An increasing body of evidence suggests that microglia are affected by ES (As reviewed by (Johnson and Kaffman, 2018)). When studying microglia, there are several parameters of relevance that can be assessed at the morphological level, including cell density, cell morphology and complexity, and the expression of phagocytic markers (e.g. CD68) as a proxy for phagocytic activity. The effect of ES on microglial cell density is not clear. MS studies have reported an increase (Delpech et al., 2016), a decrease (Saavedra et al., 2017) or no effect on microglial cell density (Roque et al., 2016). LBN exposure has however, been consistently reported not to lead to changes in microglial cell density in the hippocampus of young (Dayananda et al., 2023) and adult mice (Hoeijmakers et al., 2017; Reemst et al., 2022a), consistent with our current findings. The lack of ES-induced alterations in microglial complexity is, however, in contrast with the earlier detected reduction in microglial complexity in the hilus and the stratum lacunosum moleculare (SLM) in one of our previous studies, at roughly the same age (Reemst et al., 2022a).

This discrepancy might be due to the specific subregions analyzed, as we here assessed microglia in the DG and the CA (from which the SLM is a compartment), but not the hilus. This may have potentially diluted the effects in the SLM. Furthermore, these animals have been injected with saline, and the injection itself (either via stress or inflammation (Swan et al., 2023)) might have triggered effects in microglia, for which we cannot account when comparing across studies. Finally, we have now used a different approach for assessing microglial complexity.

The observed ES-induced increase in CD68 expression is in line with previous findings by our group (Reemst et al., 2022a; Yam et al., 2019) and others (Delpech et al., 2016; Dayananda et al., 2023). More recently, our group has also shown that expression of genes regulating phagocytosis (such as Gas6) was upregulated in the transcriptome of adult ES-exposed mice (Reemst et al., 2022a). Moreover, ES has been reported to downregulate phagocytosis of synaptosome in an ex vivo assay (Reemst et al., 2022a) as well of synaptic pruning (Dayananda et al., 2023; Bolton et al., 2022). It is intriguing that the increased expression of phagocytic markers in situ in adulthood (Hoeijmakers et al., 2017; Yam et al., 2019) would suggest functional implications opposite to those observed (Reemst et al., 2022a; Dayananda et al., 2023; Bolton et al., 2022). This might suggest that ES effects on adult microglial phagocytosis are more complex and might be highly dependent on the specific substrates. In addition, even though the current diet did not reduce the ES-induced increase in CD68 coverage, we made interesting observations regarding the correlation of microglial measures and learning score. It appeared that while the learning score did not correlate with CD68 in the CTL groups, this positively correlated with CD68 in the ES Poly group specifically, while this was negative in the ES Std group. Considering that CD68 is increased in both groups but there are different learning outcomes suggest that there might be other mechanism contributing to the beneficial effects of the diet in the ES-induced behavioral alterations.

This is the first study addressing if and how microglia alterations might contribute to the effect of polyphenol supplementation in the context of ES. In the current study, we report an increased Iba1+ cell density in the CA of and a slightly reduced cellular complexity in the DG by the Poly diet of CTL animals. While the functional implication of such increase in number of microglial cells remains to be determined it remains intriguing that the Poly diet may have different impact on brain microglia under baseline conditions versus brains previously exposed to ES. Polyphenols from grapes have been shown to improve chronic stress-induced anxiety- and depressive-like behaviors in an adult setting, where they also reversed the associated microglial activation (Yang et al., 2023). In addition, polyphenols have been shown to modulate other aspects of neuroinflammation in models of ES (Arabi et al., 2021; Chuang et al., 2013). For example, kolaviron- (Omotoso et al., 2020) and auraptene (Arabi et al., 2021) supplementation reduced MS- induced increases in glial fibrillary protein, and TLR-4 and Il-1β expression, respectively. These effects were found in younger animals, which were still on the diet when sacrificed ((Omotoso et al., 2020): P35, (Arabi et al., 2021): P60). Taken together, this suggests that the anti-inflammatory actions of polyphenols might contribute to their beneficial effects by counteracting the effect of (early-life) stress on behavior, but further studies are needed to further understand how exactly polyphenols exert their anti-inflammatory properties in the brain.

Finally, we have shown previously that nutritional intervention with anti-inflammatory N-3 PUFAs during the same timeframe as in the current study, led to a reduction of the ES-induced increase in CD68^+^ expression (Yam et al., 2019) suggesting that coffee polyphenols ameliorate ES-induced deficits via different processes.

#### 4.2.3 Limitations of the study

While our study presents some unique strengths, it also presents with some limitations. In the current study we focused on males only, as in our LBN model, ES specifically impairs cognitive functions in males, but not in females at this age (Naninck et al., 2015). We therefore choose to test the potential effectiveness of coffee polyphenols to protect against the ES-induced behavioral alterations in males. Considering the emerging evidence for the sex-dependence of the impact ES on various domains (Goodwill et al., 2019; Bale and Epperson, 2015; Reemst et al., 2022b), it will be of importance to address the potential for nutritional strategies in the context of ES also in females when focusing on other domains. In the current study we addressed a possible association between the impact of ES and diet on hippocampal cell survival and microglia however causal implication of any of the observed changes will require future investigations. Next to the mechanisms that we addressed here, other processes known to be affected by ES and polyphenols such as apoptosis (Arabi et al., 2021; Sun et al., 2021), oxidative stress (Arabi et al., 2021; Omotoso et al., 2020; Menezes et al., 2017; Zheng et al., 2015), the microbiome and the HPA-axis (Donoso et al., 2020) might be of interest for future research.

In addition, we did not assess whether caffeic or chlorogenic acids or their derivatives were indeed present in the breast milk from the dam, the peripheral tissues, or the brain of the offspring. While it is established that polyphenols can be transmitted through breast milk (Lu et al., 2021; Ríos et al., 2022), it is yet unsure what exactly is the metabolization rate of polyphenols across the blood-brain barrier (Figueira et al., 2017), thus it remains to be determined if the observed effects in the present study are direct or indirect or rather a combination of both.

## 5 Conclusion

The supplementation of polyphenols in a diet emerges as a promising avenue for nutritional intervention in the context of mitigating the detrimental cognitive effects of ES in males which are associated with microglial changes. Next to the processes that we addressed here, other processes that are known to be affected by ES might be of interest for future research. While in the current study we focused on an early time window for intervention aiming at preventing the ES-induced effects, additional time windows of intervention aiming at rescuing these will be of relevance to study in the future. Overall, our findings underscore the potential of early caffeic- and chlorogenic acid supplementation in the combatting the effect of ES.

## Funding

This project was part of the EU consortium DCogPlast ‘Diet Cognition and Plasticity’ funded by JPI-HDHL (Medical Research Council UK: MR/N030087/1; French National Research Agency.

ANR-15-HDHL-0002-05; PCIN-2015-229- MINECO); AK is funded by 10.13039/501100003246NWO Food cognition and behavior, JPI NutriCog, ZonMW-MODEM and Alzheimer Nederland; PJL is funded by 10.13039/501100010969Alzheimer Nederland, ZonMW-MODEM and the Center for Urban Mental Health from the University of Amsterdam; 10.13039/100004801ADP funded by JPI-HDHL (10.13039/501100000265Medical Research Council UK: MR/N030087/1); RGD was recipient of a “Juan de la Cierva” grant (FJCI-2015-26590) funded by MCIN.

## CRediT authorship contribution statement

**J. Geertsema:** Writing – review & editing, Writing – original draft, Visualization, Methodology, Formal analysis, Conceptualization. **M. Kratochvil:** Writing – review & editing, Formal analysis. **R. González-Domínguez:** Writing – review & editing. **S. Lefèvre-Arbogast:** Writing – review & editing. **D.Y. Low:** Writing – review & editing. **A. Du Preez:** Writing – review & editing. **H. Lee:** Writing – review & editing. **M. Urpi-Sarda:** Writing – review & editing. **A. Sánchez-Pla:** Writing – review & editing. **L. Aigner:** Writing – review & editing, Conceptualization. **C. Samieri:** Writing – review & editing, Conceptualization. **C. Andres-Lacueva:** Writing – review & editing. **C. Manach:** Writing – review & editing. **S. Thuret:** Writing – review & editing, Funding acquisition, Conceptualization. **P.J. Lucassen:** Writing – review & editing, Funding acquisition. **A. Korosi:** Conceptualization, Writing – review & editing, Writing – original draft, Funding acquisition.

## Declaration of generative AI and AI-assisted technologies in the writing process

The authors declare no use of generative AI and AI-assisted technologies in the writing process.

## Declaration of competing interest

The authors declare that they have no known competing financial interests or personal relationships that could have appeared to influence the work reported in this paper.

## Data Availability

Data will be made available on request.

## Appendix A Supplementary data

Multimedia component 1 Multimedia component 1

Supplementary Fig. 1 **ES and diet effects on bodyweight of offspring and food intake of pups and dams****.** A) ES, Polyphenol diet and age affected bodyweight throughout lifetime. B) ES and diet did not affect bodyweight gain between P9 and P21. C) ES and diet did not affect bodyweight at P42. D) ES and diet did not affect food intake in the dams between P2 and P9. E) ES and diet did not affect food intake in the dams between P9-21. F) ES and diet did not affect food intake in the offspring between P21-42. G) ES and diet did not affect food intake in the offspring between P42-49. Annotations: For two- or three-way ANOVAs, % is age effect p < 0.05, $ is ES effect p < 0.05, & is diet effect p < 0.05, ns is not significant p > 0.05. All values are Data ±SEM. Supplementary Fig. 1

Supplementary Fig. 2 **ES and diet effects on other parameters assessed in the behavioral test battery****.** A) ES and diet did not affect locomotor activity during habituation to the arenas before ORT and OLT. B) ES and diet did not affect the total exploration time for both objects during the training phase of the ORT and OLT. C) All animals had a faster acquisition time over the training period during the acquisition phase of the first and last trial combined. ES and diet did not affect the time to reach the platform in the MWM. D) All animals had a faster acquisition time over the training period during the acquisition phase of the first trials in the MWM. There was an interaction between day of training, diet, and ES. E) All animals had a faster acquisition time over the training period during the acquisition phase of the last trial in the MWM. ES and diet did not affect time to reach the platform. F) ES animals fed the Std diet spent more time in the target quadrant compared to other quadrants during the first 30 s of the probe trial in the MWM, whereas all other groups failed to perform better than chance. G) Diet reduced the latency to reach the target quadrant in the MWM, without any effect of stress. H) ES or diet did not affect distance moved during the probe trial. I) The time to reach the platform in the reversal phase of the MWM was unaffected by day of training, ES or diet. Annotations: For two- or three-way ANOVAs % is day of training effect p < 0.05, & is diet effect p < 0.05, @ is interaction effect day of training x ES x diet, * is post hoc Tukey effect p < 0.05; for one-sample *t*-test # is different from 25% p < 0.05, ns is not significant p > 0.05. All values are Data ±SEM. Supplementary Fig. 2

Supplementary Fig. 3 **ES and diet effects on other parameters assessed in the microglial analyses****.** – A) There was an interaction of diet and ES on iba1+ cell density in the hippocampus. B) There was no effect of ES or diet on Iba1+ cell density in the dorsal hippocampus. C) There was no effect of ES or diet on Iba1+ cell density in the ventral hippocampus. D) Amount of intersections plotted against the distance from the soma of hippocampal Iba1+ cells. E) ES and diet did not change the AUC of the hippocampal Sholl plots. F) ES and diet did not affect the number of primary branches of hippocampal Iba1+ cells. G) PCA plot representing all cell coordinates (shown as dots) on the first two dimensional planes PC1/PC2. The percentage of variability retained in the respective PCs is stated between brackets on the x- and y-axes. Colors indicate experimental groups. H) PCA plot representing all cell coordinates (shown as dots) on the first two dimensional planes PC1/PC2. The percentage of variability retained in the respective PCs is stated between brackets on the x- and y-axes. Colors indicate Iba1+ cell morphotypes. I) There was no effect of ES or diet on relative morphotype proportions in the hippocampus. J) ES increased CD68 coverage in the hippocampus, independent of diet. K) ES increased CD68 coverage in the dorsal hippocampus, independent of diet. L) There was no effect of stress or diet in CD68 coverage in the ventral hippocampus. M) Learning score did not correlate with Iba1+ cell density. Annotations: For two-way ANOVAs $ is ES effect p < 0.05, & is diet effect p < 0.05, ^ is interaction effect ES x Diet p < 0.05, * is post hoc Tukey effect p < 0.05. All values are Data ±SEM. Supplementary Fig. 3

## References

[bib1] An L., Zhang Y.Z., Yu N.J., Liu X.M., Zhao N., Yuan L. (2008). The total flavonoids extracted from Xiaobuxin-Tang up-regulate the decreased hippocampal neurogenesis and neurotrophic molecules expression in chronically stressed rats. Prog. Neuro-Psychopharmacol. Biol. Psychiatry.

[bib2] Arabi M., Sh N., Lorigooini Z., Sn B., Sm M., Anjomshoa M. (2021). Auraptene exerts protective effects on maternal separation stress-induced changes in behavior, hippocampus, heart and serum of mice. Int. Immunopharmacol..

[bib3] Bachiller S., Hidalgo I., Garcia M.G., Boza-Serrano A., Paulus A., Denis Q. (2022). Early-life stress elicits peripheral and brain immune activation differently in wild type and 5xFAD mice in a sex-specific manner. J. Neuroinflammation.

[bib4] Baek S.B., Bahn G., Moon S.J., Lee J., Kim K.H., Ko I.G. (2011). The phosphodiesterase type-5 inhibitor, tadalafil, improves depressive symptoms, ameliorates memory impairment, as well as suppresses apoptosis and enhances cell proliferation in the hippocampus of maternal-separated rat pups. Neurosci. Lett..

[bib5] Bale T.L., Epperson C.N. (2015). Sex differences and stress across the lifespan. Nat. Neurosci..

[bib6] Bolton J.L., Short A.K., Othy S., Kooiker C.L., Shao M., Gunn B.G. (2022). Early stress-induced impaired microglial pruning of excitatory synapses on immature CRH-expressing neurons provokes aberrant adult stress responses. Cell Rep..

[bib7] Bonapersona V., Joëls M., Sarabdjitsingh R.A. (2018). Effects of early life stress on biochemical indicators of the dopaminergic system: a 3 level meta-analysis of rodent studies. Neurosci. Biobehav. Rev..

[bib8] Carr C.P., Martins C.M.S., Stingel A.M., Lemgruber V.B., Juruena M.F. (2013). The role of early life stress in adult psychiatric disorders. J. Nerv. Ment. Dis..

[bib9] Carregosa D., Mota S., Ferreira S., Alves-Dias B., Loncarevic-Vasiljkovic N., Crespo C.L. (2021). Overview of beneficial effects of (Poly)phenol metabolites in the context of neurodegenerative diseases on model organisms. Nutrients.

[bib10] Chuang D.Y., Chan M.H., Zong Y., Sheng W., He Y., Jiang J.H. (2013). Magnolia polyphenols attenuate oxidative and inflammatory responses in neurons and microglial cells. J. Neuroinflammation.

[bib11] Corral-Jara K.F., Nuthikattu S., Rutledge J., Villablanca A., Morand C., Schroeter H. (2021). Integrated multi-omic analyses of the genomic modifications by gut microbiome-derived metabolites of epicatechin, 5-(4′-Hydroxyphenyl)-γ-Valerolactone, in TNFalpha-stimulated primary human brain microvascular endothelial cells. Front. Neurosci..

[bib12] Dayananda K.K., Ahmed S., Wang D., Polis B., Islam R., Kaffman A. (2023). Early life stress impairs synaptic pruning in the developing hippocampus. Brain Behav. Immun..

[bib13] de Vries K., Medawar E., Korosi A., Witte A.V. (2021). The effect of polyphenols on working and episodic memory in non-pathological and pathological aging: a systematic review and meta-analysis. Front. Nutr..

[bib14] Delpech J.C., Wei L., Hao J., Yu X., Madore C., Butovsky O. (2016). Early life stress perturbs the maturation of microglia in the developing hippocampus. Brain Behav. Immun..

[bib15] Diz-Chaves Y., Astiz M., Bellini M.J., Garcia-Segura L.M. (2013). Prenatal stress increases the expression of proinflammatory cytokines and exacerbates the inflammatory response to LPS in the hippocampal formation of adult male mice. Brain Behav. Immun..

[bib16] Donoso F., Egerton S., Tfs B., Fitzgerald P., Gite S., Fouhy F. (2020). Polyphenols selectively reverse early-life stress-induced behavioural, neurochemical and microbiota changes in the rat. Psychoneuroendocrinology.

[bib17] Figueira I., Garcia G., Pimpão R.C., Terrasso A.P., Costa I., Almeida A.F. (2017). Polyphenols journey through blood-brain barrier towards neuronal protection. Sci. Rep..

[bib18] Gomez-Pinilla F., Nguyen T.T. (2012). Natural mood foods: the actions of polyphenols against psychiatric and cognitive disorders. Nutr. Neurosci..

[bib19] González‐Domínguez R., Castellano‐Escuder P., Carmona F., Lefèvre‐Arbogast S., Low D.Y., Du Preez A. (2021). Food and microbiota metabolites associate with cognitive decline in older subjects: a 12‐year prospective study. Mol. Nutr. Food Res..

[bib20] Goodwill H.L., Manzano-Nieves G., Gallo M., Lee H.I., Oyerinde E., Serre T. (2019). Early life stress leads to sex differences in development of depressive-like outcomes in a mouse model. Neuropsychopharmacology.

[bib21] Green H.F., Nolan Y.M. (2014). Inflammation and the developing brain: consequences for hippocampal neurogenesis and behavior. Neurosci. Biobehav. Rev..

[bib22] Hakamata Y., Suzuki Y., Kobashikawa H., Hori H. (2022). Neurobiology of early life adversity: a systematic review of meta-analyses towards an integrative account of its neurobiological trajectories to mental disorders. Front. Neuroendocrinol..

[bib23] Herpfer I., Hezel H., Reichardt W., Clark K., Geiger J., Gross C.M. (2012). Early life stress differentially modulates distinct forms of brain plasticity in young and adult mice. PLoS One.

[bib24] Hoeijmakers L., Lucassen P.J., Korosi A. (2014). The interplay of early-life stress, nutrition, and immune activation programs adult hippocampal structure and function. Front. Mol. Neurosci..

[bib25] Hoeijmakers L., Ruigrok S.R., Amelianchik A., Ivan D., van Dam A.M., Lucassen P.J. (2017). Early-life stress lastingly alters the neuroinflammatory response to amyloid pathology in an Alzheimer's disease mouse model. Brain Behav. Immun..

[bib26] Johnson F.K., Kaffman A. (2018). Early life stress perturbs the function of microglia in the developing rodent brain: new insights and future challenges. Brain Behav. Immun..

[bib27] Juncker H.G., van Keulen B.J., Finken M.J.J., de Rooij S.R., van Goudoever J.B., Korosi A. (2022).

[bib28] Korosi A., Naninck E.F., Oomen C.A., Schouten M., Krugers H., Fitzsimons C. (2012). Early-life stress mediated modulation of adult neurogenesis and behavior. Behav. Brain Res..

[bib29] Lajud N., Roque A., Cajero M., Gutierrez-Ospina G., Torner L. (2012). Periodic maternal separation decreases hippocampal neurogenesis without affecting basal corticosterone during the stress hyporesponsive period, but alters HPA axis and coping behavior in adulthood. Psychoneuroendocrinology.

[bib30] Low D.Y., Lefèvre‐Arbogast S., González‐Domínguez R., Urpi‐Sarda M., Micheau P., Petera M. (2019). Diet‐related metabolites associated with cognitive decline revealed by untargeted metabolomics in a prospective cohort. Mol. Nutr. Food Res..

[bib31] Lu Z., Chan Y.T., Lo K.K.H., Wong V.W.S., Ng Y.F., Li S.Y. (2021). Levels of polyphenols and phenolic metabolites in breast milk and their association with plant-based food intake in Hong Kong lactating women. Food Funct..

[bib32] Lucassen P.J., Oomen C.A., Naninck E.F.G., Fitzsimons C.P., van Dam A.M., Czeh B. (2015). Regulation of adult neurogenesis and plasticity by (early) stress, glucocorticoids, and inflammation. Cold Spring Harbor Perspect. Biol..

[bib33] Lumertz F.S., Kestering-Ferreira E., Orso R., Creutzberg K.C., Tractenberg S.G., Stocchero B.A. (2022). Effects of early life stress on brain cytokines: a systematic review and meta-analysis of rodent studies. Neurosci. Biobehav. Rev..

[bib34] Menezes J., Neves B.H., Souza M., Mello-Carpes P.B. (2017). Green tea protects against memory deficits related to maternal deprivation. Physiol. Behav..

[bib35] Mirescu C., Peters J.D., Gould E. (2004). Early life experience alters response of adult neurogenesis to stress. Nat. Neurosci..

[bib36] Morris G., Gamage E., Travica N., Berk M., Jacka F.N., O'Neil A. (2021). Polyphenols as adjunctive treatments in psychiatric and neurodegenerative disorders: efficacy, mechanisms of action, and factors influencing inter-individual response. Free Radic. Biol. Med..

[bib37] Naninck E.F., Hoeijmakers L., Kakava-Georgiadou N., Meesters A., Lazic S.E., Lucassen P.J. (2015). Chronic early life stress alters developmental and adult neurogenesis and impairs cognitive function in mice. Hippocampus.

[bib38] Naninck E.F.G., Oosterink J.E., Yam K., Vries L.P., Schierbeek H., Goudoever J.B. (2017). Early micronutrient supplementation protects against early stress‐induced cognitive impairments. Faseb. J..

[bib39] Omotoso G.O., Mutholib N.Y., Abdulsalam F.A., Bature A.I. (2020). Kolaviron protects against cognitive deficits and cortico-hippocampal perturbations associated with maternal deprivation in rats. Anat. Cell Biol..

[bib40] Oomen C.A., Girardi C.E.N., Cahyadi R., Verbeek E.C., Krugers H., Joëls M. (2009). Opposite effects of early maternal deprivation on neurogenesis in male versus female rats. PLoS One.

[bib41] Pellizzon M.A., Ricci M.R. (2020). Choice of laboratory rodent diet may confound data interpretation and reproducibility. Curr. Dev. Nutr..

[bib42] Pinto C.J.G., Ávila-Gálvez M.Á., Lian Y., Moura-Alves P., Nunes dos Santos C. (2023). Targeting the aryl hydrocarbon receptor by gut phenolic metabolites: a strategy towards gut inflammation. Redox Biol..

[bib43] Quideau S., Deffieux D., Douat‐Casassus C., Pouységu L. (2011). Plant polyphenols: chemical properties, biological activities, and synthesis. Angew. Chem. Int. Ed..

[bib44] Reemst K., Kracht L., Kotah J.M., Rahimian R., van Irsen A.A.S., Sotomayor G.C. (2022). Early-life stress lastingly impacts microglial transcriptome and function under basal and immune-challenged conditions. Transl. Psychiatry.

[bib45] Reemst K., Ruigrok S.R., Bleker L., Naninck E.F.G., Ernst T., Kotah J.M. (2022). Sex-dependence and comorbidities of the early-life adversity induced mental and metabolic disease risks: where are we at?. Neurosci. Biobehav. Rev..

[bib46] Rice C.J., Sandman C.A., Lenjavi M.R., Baram T.Z. (2008). A novel mouse model for acute and long-lasting consequences of early life stress. Endocrinology.

[bib47] Ríos J., Valero-Jara V., Thomas-Valdés S. (2022). Phytochemicals in breast milk and their benefits for infants. Crit. Rev. Food Sci. Nutr..

[bib48] Roque A., Ochoa-Zarzosa A., Torner L. (2016). Maternal separation activates microglial cells and induces an inflammatory response in the hippocampus of male rat pups, independently of hypothalamic and peripheral cytokine levels. Brain Behav. Immun..

[bib49] Saavedra L.M., Fenton Navarro B., Torner L. (2017). Early life stress activates glial cells in the Hippocampus but attenuates cytokine secretion in response to an immune challenge in rat pups. Neuroimmunomodulation.

[bib50] Short A.K., Baram T.Z. (2019). Early-life adversity and neurological disease: age-old questions and novel answers. Nat. Rev. Neurol..

[bib51] Sun Q., Jia N., Ren F., Li X. (2021). Grape seed proanthocyanidins improves depression-like behavior by alleviating oxidative stress and NLRP3 activation in the hippocampus of prenatally-stressed female offspring rats. J. Histotechnol..

[bib52] Swan J., Boyer S., Westlund K., Bengtsson C., Nordahl G., Törnqvist E. (2023). Decreased levels of discomfort in repeatedly handled mice during experimental procedures, assessed by facial expressions. Front. Behav. Neurosci..

[bib53] Tuck C.J., De Palma G., Takami K., Brant B., Caminero A., Reed D.E. (2020). Nutritional profile of rodent diets impacts experimental reproducibility in microbiome preclinical research. Sci. Rep..

[bib54] van Weering H.R.J., Nijboer T.W., Brummer M.L., Boddeke E.W.G.M., Eggen B.J.L. (2023). Microglia morphotyping in the adult mouse <scp>CNS</scp> using hierarchical clustering on principal components reveals regional heterogeneity but no sexual dimorphism. Glia.

[bib55] Vauzour D. (2017). Polyphenols and brain health. OCL.

[bib56] Walker C.D., Bath K.G., Joels M., Korosi A., Larauche M., Lucassen P.J. (2017). Chronic early life stress induced by limited bedding and nesting (LBN) material in rodents: critical considerations of methodology, outcomes and translational potential. Stress.

[bib57] Wang D., Wang T., Zhang Z., Li Z., Guo Y., Zhao G. (2022). Recent advances in the effects of dietary polyphenols on inflammation in vivo: potential molecular mechanisms, receptor targets, safety issues, and uses of nanodelivery system and polyphenol polymers. Curr. Opin. Food Sci..

[bib58] Yam K., Schipper L., Reemst K., Ruigrok S.R., Abbink M.R., Hoeijmakers L. (2019). Increasing availability of ω‐3 fatty acid in the early‐life diet prevents the early‐life stress‐induced cognitive impairments without affecting metabolic alterations. Faseb. J..

[bib59] Yang E.J., Frolinger T., Iqbal U.H., Murrough J., Pasinetti G.M. (2023). The bioactive dietary polyphenol preparation alleviates depression and anxiety-like behaviors by modulating the regional heterogeneity of microglia morphology. Mol. Nutr. Food Res..

[bib60] Zheng A., Li H., Cao K., Xu J., Zou X., Li Y. (2015). Maternal hydroxytyrosol administration improves neurogenesis and cognitive function in prenatally stressed offspring. J. Nutr. Biochem..

[bib61] Zheng X., Cheng Y., Chen Y., Yue Y., Li Y., Xia S. (2019). Ferulic acid improves depressive-like behavior in prenatally-stressed offspring rats via anti-inflammatory activity and HPA Axis. Int. J. Mol. Sci..

