# Supplementary figures and images for: Coffee polyphenols ameliorate early-life stress-induced cognitive deficits in male mice
Source: Neurobiol Stress. 2024 May 15;31:100641. doi: 10.1016/j.ynstr.2024.100641 (PMC11140806; doi:10.1016/j.ynstr.2024.100641)

**A**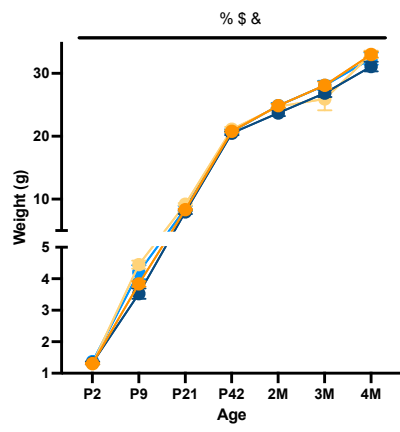**B**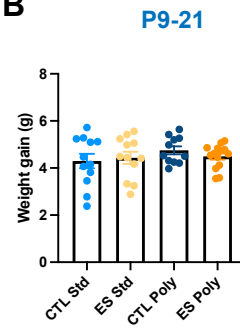**C**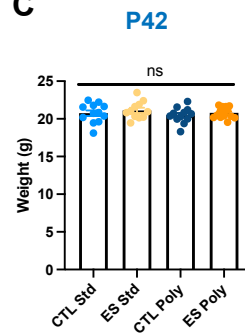**D**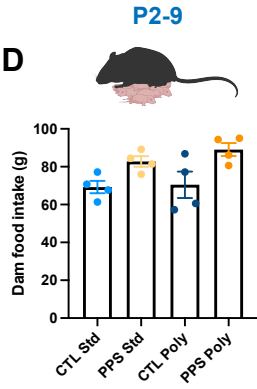**E**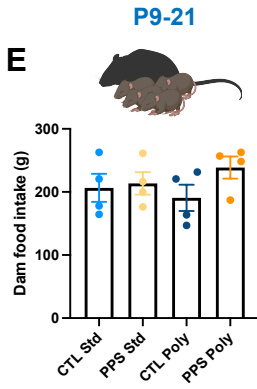**F**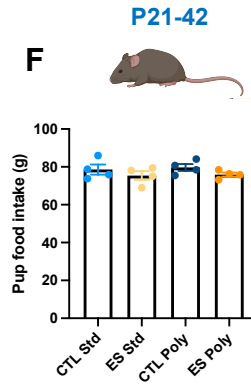**G**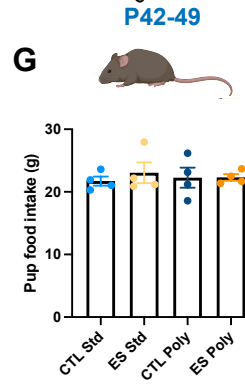

Supplement: Supplementary Fig. 1 — ES and diet effects on bodyweight of offspring and food intake of pups and dams. A) ES, Polyphenol diet and age affected bodyweight throughout lifetime. B) ES and diet did not affect bodyweight gain between P9 and P21. C) ES and diet did not affect bodyweight at P42. D) ES and diet did not affect food intake in the dams between P2 and P9. E) ES and diet did not affect food intake in the dams between P9-21. F) ES and diet did not affect food intake in the offspring between P21-42. G) ES and diet did not affect food intake in the offspring between P42-49. Annotations: For two- or three-way ANOVAs, % is age effect p < 0.05, $ is ES effect p < 0.05, & is diet effect p < 0.05, ns is not significant p > 0.05. All values are Data ±SEM. [file mmc2.pdf]

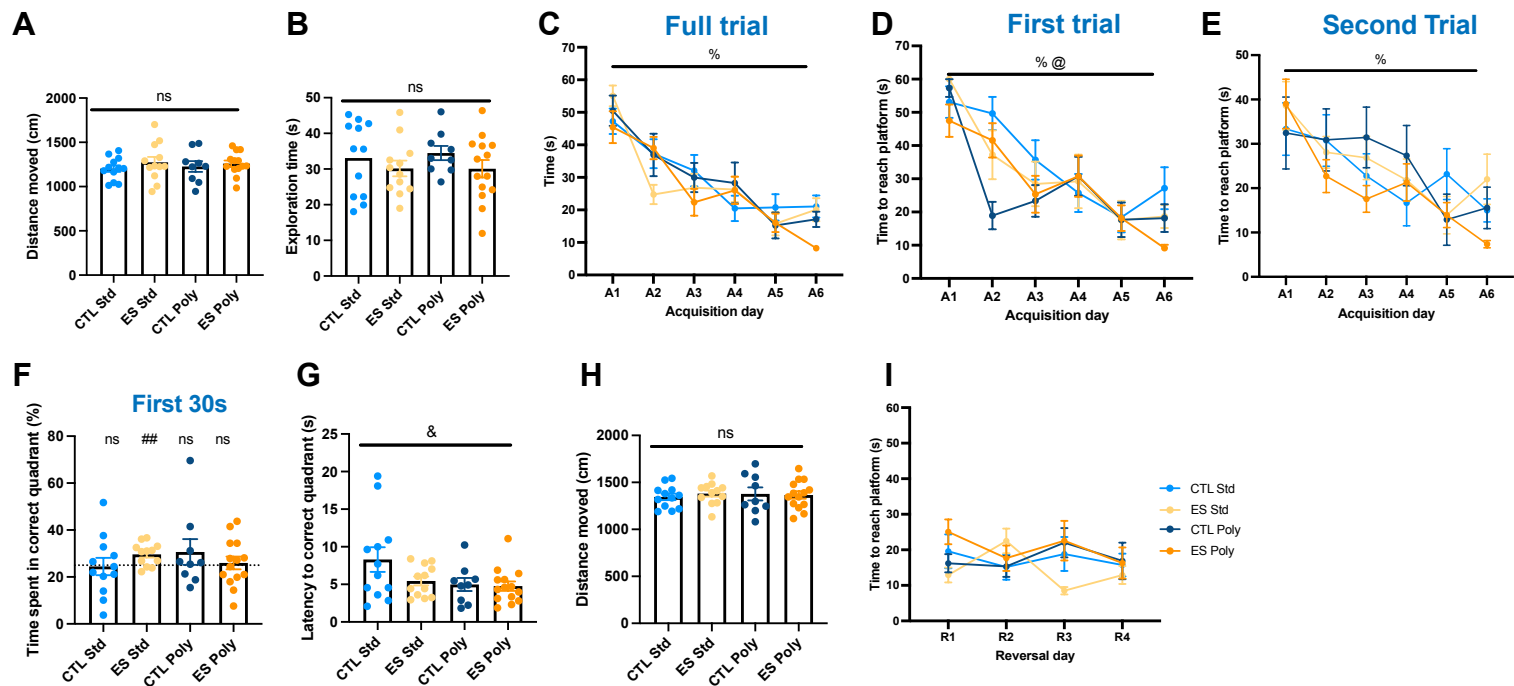

Supplement: Supplementary Fig. 2 — ES and diet effects on other parameters assessed in the behavioral test battery. A) ES and diet did not affect locomotor activity during habituation to the arenas before ORT and OLT. B) ES and diet did not affect the total exploration time for both objects during the training phase of the ORT and OLT. C) All animals had a faster acquisition time over the training period during the acquisition phase of the first and last trial combined. ES and diet did not affect the time to reach the platform in the MWM. D) All animals had a faster acquisition time over the training period during the acquisition phase of the first trials in the MWM. There was an interaction between day of training, diet, and ES. E) All animals had a faster acquisition time over the training period during the acquisition phase of the last trial in the MWM. ES and diet did not affect time to reach the platform. F) ES animals fed the Std diet spent more time in the target quadrant compared to other quadrants during the first 30 s of the probe trial in the MWM, whereas all other groups failed to perform better than chance. G) Diet reduced the latency to reach the target quadrant in the MWM, without any effect of stress. H) ES or diet did not affect distance moved during the probe trial. I) The time to reach the platform in the reversal phase of the MWM was unaffected by day of training, ES or diet. Annotations: For two- or three-way ANOVAs % is day of training effect p < 0.05, & is diet effect p < 0.05, @ is interaction effect day of training x ES x diet, * is post hoc Tukey effect p < 0.05; for one-sample t-test # is different from 25% p < 0.05, ns is not significant p > 0.05. All values are Data ±SEM. [file mmc3.pdf]

**A**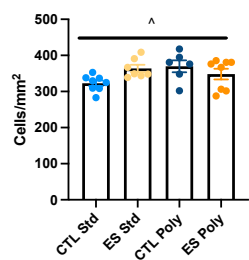**B**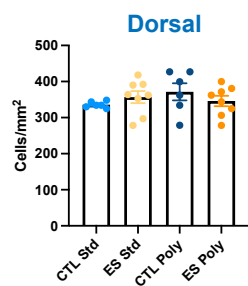**C**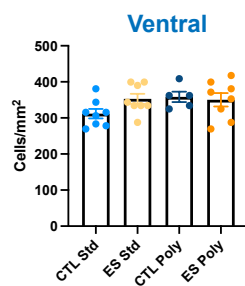**D**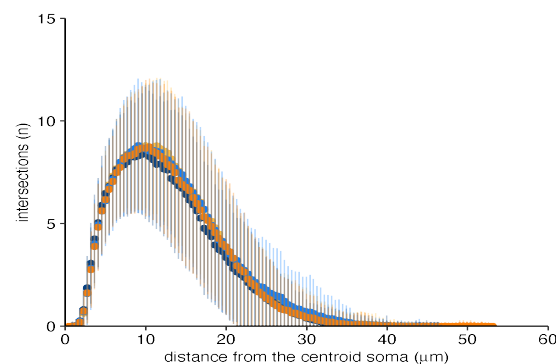**E**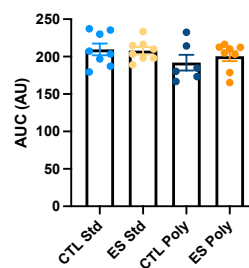**F**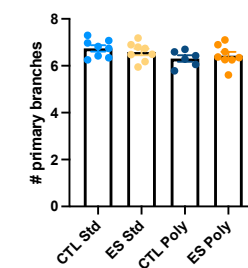**G**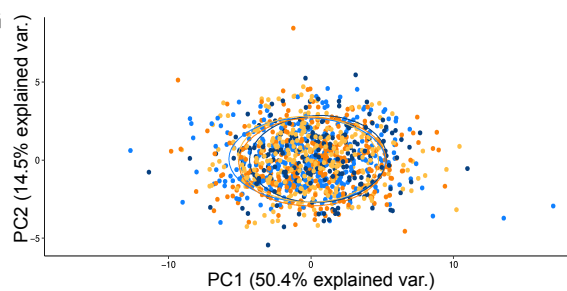**I**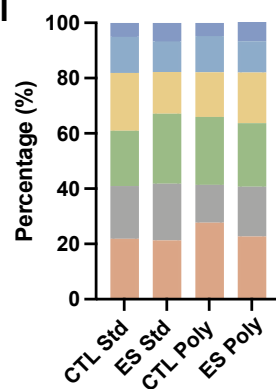**J**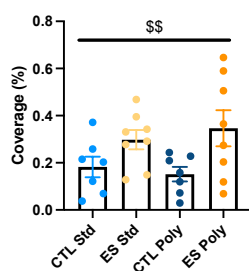**K**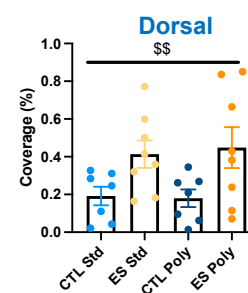**L**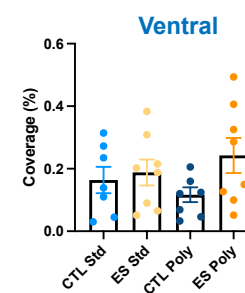**M**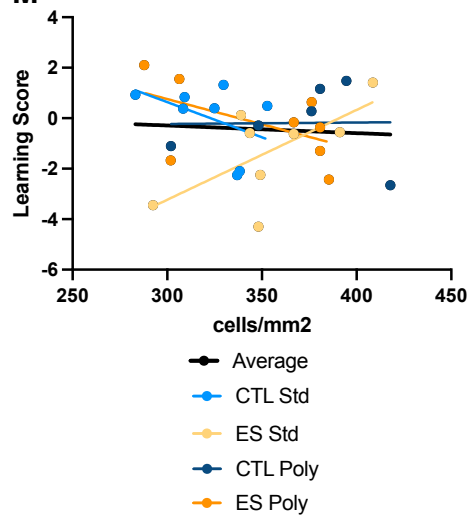

Supplement: Supplementary Fig. 3 — ES and diet effects on other parameters assessed in the microglial analyses. – A) There was an interaction of diet and ES on iba1+ cell density in the hippocampus. B) There was no effect of ES or diet on Iba1+ cell density in the dorsal hippocampus. C) There was no effect of ES or diet on Iba1+ cell density in the ventral hippocampus. D) Amount of intersections plotted against the distance from the soma of hippocampal Iba1+ cells. E) ES and diet did not change the AUC of the hippocampal Sholl plots. F) ES and diet did not affect the number of primary branches of hippocampal Iba1+ cells. G) PCA plot representing all cell coordinates (shown as dots) on the first two dimensional planes PC1/PC2. The percentage of variability retained in the respective PCs is stated between brackets on the x- and y-axes. Colors indicate experimental groups. H) PCA plot representing all cell coordinates (shown as dots) on the first two dimensional planes PC1/PC2. The percentage of variability retained in the respective PCs is stated between brackets on the x- and y-axes. Colors indicate Iba1+ cell morphotypes. I) There was no effect of ES or diet on relative morphotype proportions in the hippocampus. J) ES increased CD68 coverage in the hippocampus, independent of diet. K) ES increased CD68 coverage in the dorsal hippocampus, independent of diet. L) There was no effect of stress or diet in CD68 coverage in the ventral hippocampus. M) Learning score did not correlate with Iba1+ cell density. Annotations: For two-way ANOVAs $ is ES effect p < 0.05, & is diet effect p < 0.05, ^ is interaction effect ES x Diet p < 0.05, * is post hoc Tukey effect p < 0.05. All values are Data ±SEM. [file mmc4.pdf]
